# First direct observations of atmospheric sputtering at Mars

**DOI:** 10.1126/sciadv.adt1538

**Published:** 2025-05-28

**Authors:** Shannon M. Curry, Takuya Hara, Janet G. Luhmann, Francois Leblanc, Rebecca Jolitz, David Mitchell, Ronan Modolo, David A. Brain, Jared Espley, Mehdi Benna, Jasper Halekas

**Affiliations:** ^1^ Laboratory for Atmospheric and Space Physics, University of Colorado Boulder, Boulder, CO 80304, USA.; ^2^ Space Sciences Laboratory, University of California at Berkeley, Berkeley, CA 94720.; ^3^ LATMOS-IPSL/CNRS, Guyancourt F-78280, France.; ^4^ NASA Goddard Space Flight Center, Greenbelt, MD 20771, USA.; ^5^ Department of Physics and Astronomy, University of Iowa, Iowa City, IA 52242 USA.

## Abstract

Billions of years ago, Mars’ ability to sustain liquid water waned as the solar wind and radiation began to erode the atmosphere. Sputtering is an atmospheric escape process that may have been dominant during earlier epochs of our Sun according to isotopic evidence, but is difficult to detect under current solar conditions. Using over 9 years of data from the Mars Atmosphere and Volatile Evolution mission, we present the first observations of present-day sputtering in the martian upper atmosphere. By correlating argon densities with solar electric fields, we find that sputtered rates of argon are over four times higher than model predictions. We also present evidence of enhanced sputtering during a solar storm, offering a glimpse at more intense conditions in the early solar system. Observationally establishing the role of sputtering in the loss of Mars’ atmosphere is critical to understanding the conditions that allowed liquid water to exist on the martian surface and the implications for habitability.

## Teaser

Mars Atmosphere and Volatile Evolution (MAVEN) made the first direct observations of atmospheric sputtering in Mars’ atmosphere.

## INTRODUCTION

Abundant evidence indicates that liquid water was stable on the martian surface billions of years ago; however, this would have required a much higher atmospheric surface pressure than today. Understanding how Mars’ atmosphere eroded and evolved over time is a critical aspect of understanding how and when liquid water existed on the surface. Atmospheric escape at Mars is substantially different from that at Earth because Mars lacks a global dynamo magnetic field. This creates a scenario where the solar wind can directly interact with the upper atmosphere. Neutral constituents in the upper atmosphere are ionized and can be directly accelerated away from the planet and escape, or they can be accelerated toward the planet and precipitate back into the atmosphere. When the ions precipitate, they collide with neutrals in the upper atmosphere and can transfer enough energy for the neutrals to exceed their escape velocity, a process known as atmospheric sputtering (*1*). While the photochemical and Jeans escape processes dominate present-day martian atmospheric loss, sputtering has been proposed to be the dominant escape process during the early epochs of our solar system when the solar activity and extreme ultraviolet (EUV) intensities were much higher than the present day (*2*–*4*).

The process of atmospheric sputtering at Mars has never previously been observed. Many models of present-day sputtering have focused on O, O_2_, and CO_2_, but these constituents are highly reactive and dominated by photochemistry, so it is extremely difficult to observationally resolve the sputtered versus highly variable photochemical components of oxygen and carbon (*5*, *6*). However, argon is an excellent marker for sputtering and atmospheric evolution because it is an inert noble gas that is heavy and not easily ionized, so the processes to remove argon are limited. In addition, studies of isotopic fractionation in ^38^Ar/^36^Ar ratios indicate that the lighter argon isotopes have been preferentially removed over heavier ones (*4*). Only the atmospheric sputtering process would produce an enrichment of heavy argon isotopes of this magnitude since a process such as hydrodynamic outflow is inefficient at fractionating isotopes with a small mass difference, i.e., preferentially removing ^36^Ar over ^38^Ar (*7*). Furthermore, studies based on isotopic fractionation suggest that sputtering would have been dominant at Mars billions of years ago (*2*).

### Difficulty of measuring sputtering

**Fig. 1.**
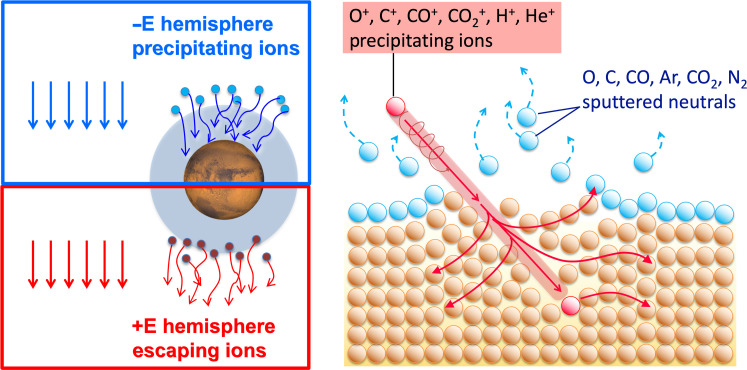
Sputtering driven by the solar wind motional electric field. (**Left**) An illustration of the ***−E*** and ***+E*** hemispheres resulting from the solar wind motional electric field defined by *E* = −*U* × *B*, where *U* is the solar wind velocity and *B* is the IMF. Ions precipitate into the martian atmosphere in the ***−E*** hemisphere and escape in the ***+E*** hemisphere. (**Right**) An illustration of sputtering: A heavy ion (red) precipitates into the neutral upper atmosphere and transfers momentum to the neutral particles (orange) in the upper atmosphere via collisions. Some of the newly energized neutral atoms and molecules (blue) can subsequently escape to space.

There are numerous challenges to observing atmospheric sputtering at Mars. Sputtering occurs when ions are accelerated by the solar wind electric field and precipitate into the martian upper atmosphere, colliding with neutrals, a fraction of which exceed the escape velocity. Figure 1 illustrates the process of sputtering; a successful observation requires simultaneous measurements of (i) the catalyst, either the precipitating ions or the solar wind electric field, and (ii) the sputtered neutrals. Moreover, to evaluate the effects of sputtering globally, observations are needed across the dayside and nightside of Mars and throughout the upper atmosphere (~200 to 400 km).

Currently, the Mars Atmosphere and Volatile Evolution (MAVEN) spacecraft is the only orbiter that has the instrument suite and orbit to observe the sputtering process and its effects in situ. Early in the MAVEN mission, a number of studies investigated precipitating ions but were not able to observe sputtered neutrals. Using MAVEN data, Leblanc *et al.* (*8*) observed heavy ion precipitation that indicated that sputtering should certainly be occurring at Mars. Hara *et al.* (*9*) and Brain *et al.* (*10*) showed that heavy ion precipitation is indeed organized with respect to the solar wind motional electric field: $\vec{E}=-\vec{U}\times \vec{B}$ , which is the cross product of the solar wind velocity, *U*, and the interplanetary magnetic field (IMF), *B*. However, at the time of these ion precipitation studies, no clear simultaneous signature of the sputtered neutrals could be identified because of instrument pointing, spacecraft coverage, orbital constraints, and the highly variable nature of the upper martian atmosphere (*11*–*14*). Until now, no study has instead organized the neutral upper atmosphere observations by the solar wind convection electric field.

To correlate the solar wind motional electric field with sputtered neutrals, simultaneous observations of the electric fields and neutrals are necessary. As seen in the left panel of Fig. 1, ion precipitation (and subsequent sputtering) occurs when the electric field is configured to point radially into the planet, referred to as the “***−E*** hemisphere.” Conversely, the “***+E*** hemisphere” is when the electric field points radially away from the planet and accelerates ions outward to escape (*8*, *10*, *15*, *16*). We calculate the electric field by the cross product of the solar wind velocity and IMF.

In addition to the electric field, simultaneous measurements of the sputtered byproduct ^40^Ar are necessary. While high-altitude argon has been observed, earlier investigations have not been able to decipher whether the argon was produced thermally, via dissociative recombination (DR; photochemically) or via sputtering (*12*, *14*). To identify the sputtered component of argon, we need to know what altitude the sputtered component will dominate. Here, we use the HELIOSARES simulation, which is composed of a hybrid simulation (LatHyS) and an exospheric model (EGM). LatHyS is a self-consistent hybrid simulation, treating ions as particles and electrons as a fluid, and is used to characterize the three-dimensional plasma and electromagnetic environment at Mars, including the solar wind and IMF (*17*, *18*). We used the average solar wind density and IMF values as inputs to LatHyS to generate ion precipitation spectra to feed into the EGM. The EGM uses neutral collisional physics, solar EUV, and seasons to take the precipitation spectra as an input and calculate the sputtering. For our results, we used equinox values for EUV.

**Fig. 2.**
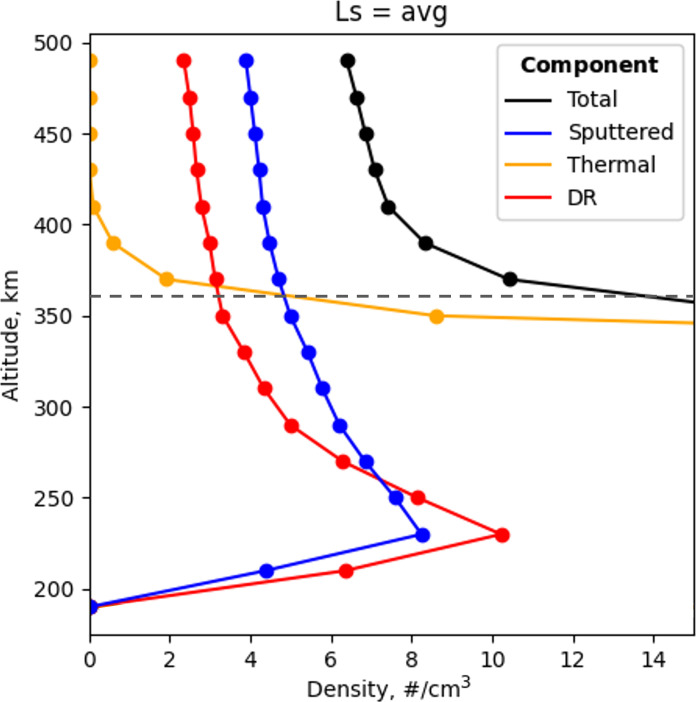
Simulated argon densities in the martian upper atmosphere. Argon densities as a function of altitude from the EGM model averaged over four seasons over the nightside [solar zenith angle (SZA) of 110° to 180°] of Mars. Total ^40^Ar densities are in black, with each component colored by gold for thermal, blue for sputtering, and red for DR. The exospheric model (EGM) predictions for argon density show that sputtering dominates above ~360 km.

To identify the altitude where sputtering dominates, the EGM model calculates the argon density for three components: (i) thermal component, characterized by a small-scale height, and the nonthermal components, (ii) DR (photochemistry) and (iii) sputtering, characterized by large-scale heights. Leblanc *et al.* (*14*, *17*) used the EGM model to show that sputtering dominates on the nightside above ~360 km (dashed line), while thermal processes dominate on the nightside below ~360 km and DR dominates on the dayside. Figure 2 illustrates the EGM simulations of argon density versus altitude for the thermal, DR, and sputtered components averaged over all martian seasons (*14*). These modeling results indicate that sputtering of ^40^Ar is dominant above ~360 km (*17*).

Last, the local time of MAVEN’s periapsis—defined by the solar zenith angle (SZA)—has a substantial influence on measured argon density. To remove these diurnal effects, the simultaneous measurements of the solar wind, IMF, and high-altitude argon must be made across all SZAs (from the dayside to the nightside).

## RESULTS

Using the Mars-Solar electric field (MSE) frame, we compared measured argon abundances in the ***−E*** and ***+E*** hemispheres from 250 to 300 km (left) and 350 to 400 km (right) over 16 SZA bins. The median ^40^Ar densities and upper and lower quartiles are shown in the top panel, followed by the ratio of the ***−E/+E*** argon abundances (log scale) in the middle panel and number of samples in the bottom panel.

**Fig. 3.**
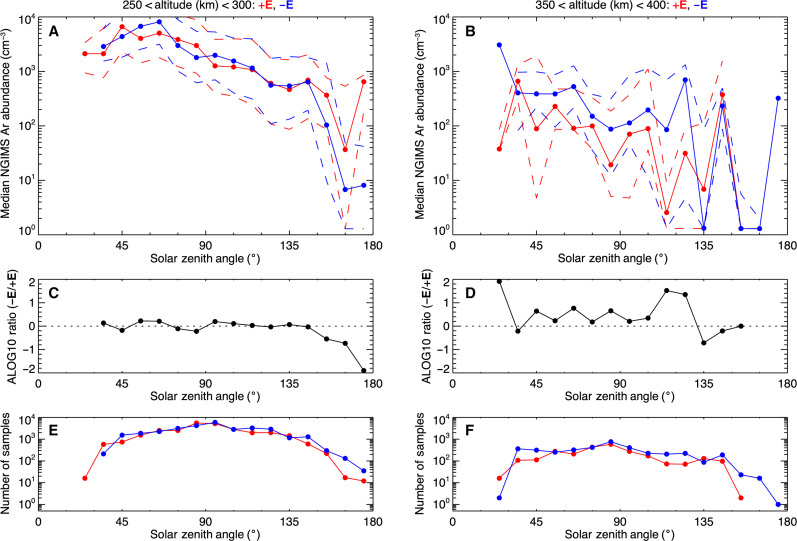
Argon density in the Mars-Solar electric (MSE) coordinate frame. (**A**) Argon abundance observed by neutral gas and ion mass spectrometer (NGIMS) from 2015 to 2024 in ***+E*** (red) and ***−E*** (blue) hemispheres as a function of the solar zenith angle (SZA) (in degrees) with altitudes between 250 and 300 km. (**B**) Same as (A), plotted between 350 and 400 km. The median values in (A) and (B) are shown by solid curves, and the dashed curves are their upper and lower quartiles. (**C**) The ratio of argon density in the ***−E*** to ***+E*** hemisphere as a function of the SZA, which is obtained from the blue solid curve divided by the red solid curve from 250 to 300 km. (**D**) Same as (C), plotted from 350 to 400 km. (**E**) The number of NGIMS observations in ***+E*** (red) and ***−E*** (blue) hemispheres as a function of the SZA from 250 to 300 km and (**F**) from 350 to 400 km.

Figure 3 illustrates that above 350 km, the ***−E*** hemisphere has higher argon densities than the ***+E*** hemisphere, while at 250 to 300 km, it does not. This result is consistent with model predictions of sputtering occurring at high altitudes and data studies of the ^36^Ar/^38^Ar isotopes, indicating that lighter argon isotopes have been preferentially removed (*4*, *14*, *19*).

## DISCUSSION

While the quartiles for the ***−E*** and ***+E*** hemispheric argon densities in Fig. 3 are large, we performed three statistical tests to validate that the signal at 350 to 400 km is statistically significant. Those tests were as follows:

1) Chi-square (χ^2^) test. A statistical test to see whether a null hypothesis (if a result is actually a random coincidence) is true. We assumed the null hypothesis to be that the argon density is randomly distributed. Using the ***−E*/*+E*** argon density ratio at 250 to 300 km and 250 to 400 km, we calculated the *P* value (the probability that the null hypothesis is true) to be 0.79%. That is, we find that there is only a 0.79% chance that the argon density at 350 to 400 km is randomly higher in the ***−E*** hemisphere rather than the *+****E*** hemisphere.

2) Spearman’s rank-order correlation test. A nonparametric test that measures the association between two ranked variables with a correlation coefficient between −1 and 1. We calculated the Spearman correlation coefficient for the ***−E/+E*** argon density ratio to be −0.044. This suggests that in the MSE frame, the argon densities at 250 to 300 km are not correlated with the argon densities at 350 to 400 km (thus, different physics are at play).

3) Mann-Whitney *U* test. A nonparametric test to assess whether two sampled variables are likely to derive from the same population. We calculated a *U* value for the ***−E/+E*** argon density ratio to be 47, which is less than the critical value of 59. This indicates that we can reject the null hypothesis that the two groups are equal and accept the hypothesis that the argon abundances at low and high altitudes are statistically different and not from the same population.

These three statistical tests demonstrate that the enhanced argon in the MSE frame at high altitudes is not a coincidence, nor associated with, nor from the same population as the argon densities from the lower altitudes. Simply put, the argon densities at higher and lower altitudes in the MSE frame are inherently different. Additional details of each statistical test are provided in the Supplementary Materials. Thus, while the signature of present-day sputtering is indeed faint as models have predicted, we find that high-altitude ^40^Ar is controlled by sputtering via electric fields driven by the sun.

This result also eliminates other possible physical processes that are known to increase the scale height of neutral and charged particles in the collisional regime, such as gravity waves, wave heating, or currents, since those processes would heat both the upper atmosphere and exosphere, not preferentially operate at 350 to 400 km over 250 to 300 (*13*, *20*–*22*). While there are SZA bins in the ***−E/+E*** ratio at 350 to 400 km that show a ratio of zero or less than zero, some of the data in those specific bins (135° and 155°) were observed during high dust activity and had far fewer data points than better sampled SZAs near the terminator. Even when the dust opacity is low, residual heating from dust activity during dust season may increase argon scale heights (*22*, *23*). Nonetheless, the data show that argon densities above 350 km have a statistically significant preference for the **−E** hemisphere, a clear and otherwise unexplainable signature of sputtering.

The sputtered argon yield is calculated from the third panel by averaging the ***−E/+E*** argon density ratio over all SZAs, which results in a 9.8 factor increase in argon density at 350 to 400 km. The HELIOSARES-EGM model predicts a factor of 2.2 increase at 400 km and a related escape rate of 4.9 × 10^22^ Ar/s for nominal solar wind conditions (*17*). We calculate the sputtered escape rate for argon to be 2.1× 10^23^ Ar/s. If the yield increase is constantly higher (a factor of 4.4× higher than model predictions), then the escape rates for other main species are: 1.2 × 10^24^ O/s, 1.6 × 10^23^ CO/s, 9.9 × 10^23^ CO_2_/s, 3.2 × 10^23^ C/s, 1.2 × 10^24^ N_2_/s, and 1.0 × 10^24^ N/s. The difference in the observed yield could be due to solar wind variability in the data that was not included in the simulated predictions, including interplanetary coronal mass ejections (ICMEs) and other space weather events that occurred during the 10-year period. ICMEs are known to increase both sputtering and ion precipitation fluxes by up to an order of magnitude (*24*–*27*). On the basis of studies in (*28*) applied over time, the cumulative effect of sputtering could increase the total erosion of the atmosphere by up to 15 to 30% with respect to oxygen and carbon depending on whether the martian atmosphere is source or energy limited.

### Case study

**Fig. 4.**
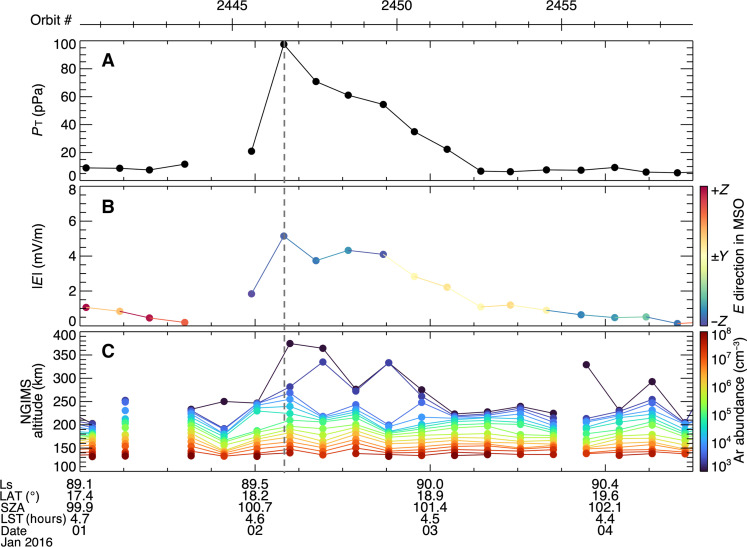
A case study of atmospheric sputtering occurring during an interplanetary coronal mass ejection (ICME) . (**A**) Upstream solar wind total perpendicular pressure (in picopascals), (**B**) electric field magnitude (in millivolts per meter) color-coded by vector direction, and (**C**) the ^40^Ar abundance altitude profile (in cubic centimeters). The horizontal bar on the top illustrates the orbit number, and dashed line marks the arrival of the ICME shock. Ls, seasonal longitude; LAT, latitude of periapsis; SZA, solar zenith angle; LST, local solar time.

Solar storms, which include solar wind density and velocity enhancements, have been shown to increase precipitating and escaping ion fluxes at Mars by both increasing the solar wind convection electric field and exposing more of the upper atmosphere to the solar wind (*29*, *30*); models have shown that the increase in precipitating ion fluxes will increase the flux of sputtered neutrals (*6*). Figure 4 illustrates the response of the martian atmosphere to an ICME that arrived at Mars in January of 2016 and drove a marked enhancement in sputtered ^40^Ar. We use the total perpendicular pressure, a commonly used diagnostic for the strength of the event, defined by $P_{\text{TOT}}=\frac{B^{2}}{2\mu_{0}}+n_{i}kT_{i}+n_{e}kT_{e}$ , where *B* is the magnetic field, *n* is the ion or electron density, and *T* is the ion or electron temperature (*31*). During the ICME, the total perpendicular pressure in the top panel increased by nearly an order of magnitude, and the solar wind motional electric field increased by a factor of almost 8. During the peak of the ICME (2 January 2016/00:00 to 2 January 2016/18:00), the electric field has a strong −*Z*_MSO_ component, and the spacecraft is in the northern hemisphere (17° to 20° North), which makes this an ideal location in the ***−E*** hemisphere for the spacecraft to observe a solar storm and sputtering simultaneously. The bottom panel of Fig. 4 shows that ^40^Ar is markedly enhanced as the shock front of the ICME reaches Mars just after 2 January 2016/04:00, designated by the dashed line. The enhancement of the argon density was 2.2 on a log scale, resulting in an increase by over two orders of magnitude, showing very good agreement with (*32*). Additional examples of sputtered argon during ICMEs are included in the Supplementary Materials, figs. S1 and S2.

In conclusion, we conducted an analysis of over 9 years of MAVEN data to determine that sputtering is an active atmospheric escape process at Mars today. Sputtering is elusive to observe under present conditions but is critical to quantify because it may have been the main pathway to erode the once thick martian atmosphere, previously capable of sustaining liquid water, to the thin, dry atmosphere observed today. Sputtering is difficult to observe because constituents in Mars’ atmosphere, such as oxygen, are often highly reactive and dominated by photochemistry so discerning whether it is the product of a sputtered or photochemical process is challenging. To address this, we analyzed argon because it is an inert, noble gas, whose isotopic markers show that lighter isotopes have been preferentially removed over heavy ones—a signature that only sputtering can produce. Using an innovative method of mapping argon in the solar wind–based MSE coordinate system, we show that argon is enhanced at high altitudes in the regions where heavy ions precipitate into the atmosphere and induce sputtering. We find that atmospheric sputtering today is over four times higher than previous predictions and that a solar storm can substantially increase the sputtered yield. Our results confirm that sputtering is occurring on present-day Mars and could have been the main pathway for atmospheric escape at Mars during the early epochs of our solar system when the solar activity and EUV intensities were much higher. These results provide a substantial step toward observationally establishing sputtering’s role in the loss of Mars’ atmosphere and, hence, in determining the history of water and those implications for habitability over time.

## MATERIALS AND METHODS

**Fig. 5.**
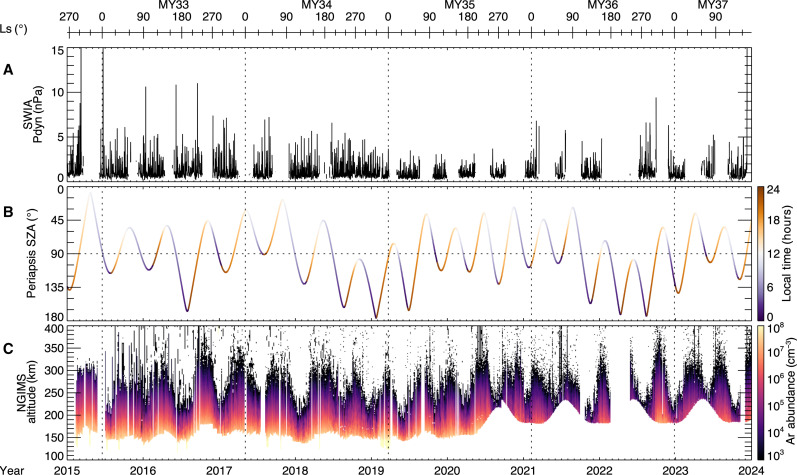
A time series of MAVEN observations between 2015 and 2024. (**A**) Upstream solar wind dynamic pressure (Pdyn; in nanopascals), (**B**) periapsis SZA color-coded by local time (in degrees and hours), and (**C**) the argon abundance altitude profile (in cubic centimeters). The horizontal bar on the top illustrates the solar longitude (Ls) characterizing the martian season (in degrees) with vertical dotted lines depicting the martian new year (MY).

Figure 5 illustrates the full mission dataset that we used to identify sputtering signatures by means of a statistical analysis of simultaneous observations of the solar wind and high-altitude argon from 2014 to 2024 across the dayside and nightside of Mars from 200 to 400 km. To determine whether the observed argon (^40^Ar), an inert noble gas, has preferentially higher densities in the ***−E*** hemisphere than the ***+E*** hemisphere, we analyzed its densities in the MSE coordinate system. The MSE coordinate system describes the frame where *X*_MSE_ is directed sunward, *Z*_MSE_ aligns with the solar wind motional electric field, and *Y*_MSE_ completes the right-hand system. To construct the MSE coordinate system, we used MAVEN’s solar wind ion analyzer (SWIA) and magnetometer (MAG), which observe the upstream solar wind and IMF conditions, respectively (*33*, *34*). As shown in Fig. 5, MAVEN does not always measure the upstream solar wind conditions due to its orbital precession, so we only use the time periods in which direct solar wind measurements are available and require the difference of the IMF clock angle between the inbound and outbound segments to be less than 45° to ensure an accurate frame transformation from the Mars-centered solar orbital (MSO) to MSE coordinates.

We statistically analyzed the ^40^Ar data between 2015 and 2024 using the neutral gas and ion mass spectrometer (NGIMS) level 2 (L2) closed source neutral version 8 data only during MAVEN’s inbound segments with a quality flag of both inbound verified (IV) and inbound unverified (IU); the bottom panel of Fig. 5 shows a time series of ^40^Ar data used in this study (*35*, *36*). While seasons and dust can heat the atmosphere and increase scale heights of all species (*37*–*39*), longer dust storms would not preferentially influence argon density enhancements in the MSE coordinate system because the upstream solar wind conditions are completely independent from dust and vary on the scale of hours (*40*). Only data during one regional dust storm (less than 3 days) was removed because it limited time periods the spacecraft sampled specific SZAs.
