# Supplementary material for: First direct observations of atmospheric sputtering at Mars
Source: Sci Adv. 2025 May 28;11(22):eadt1538. doi: 10.1126/sciadv.adt1538 (PMC12118633; doi:10.1126/sciadv.adt1538)
Supplement: Supplementary file 1 — Supplementary Text Figs. S1 and S2 [file sciadv.adt1538_sm.pdf]

Science Advances
AAAS

Supplementary Materials for

First direct observations of atmospheric sputtering at Mars

Shannon M. Curry *et al.*

Corresponding author: Shannon M. Curry, shannon.curry@colorado.edu

Sci. Adv. **11**, eadt1538 (2025)
DOI: 10.1126/sciadv.adt1538

This PDF file includes:

Supplementary Text
Figs. S1 and S2

Supplementary Text

Results: argon statistics in the MSE frame

While the quartiles for the -E and +E hemispheric argon densities are large, we performed three robust statistical tests to validate that the sputtering signal at 350-400km is statistically significant. These tests effectively ask whether argon in the -E hemisphere at 350-400km appears to be higher by chance at almost all solar zenith angles. Because the distribution of the errors is unknown, we used three non-parametric statistical tests:

1. **Chi-square (χ^2) test**: a non-parametric test that compares the observed and expected results if a null hypothesis were true; specifically, it identifies whether a disparity between the observed and expected data is due to chance or to a correlation between the variables. It also assumes that the errors are Gaussian. We assumed the null hypothesis here to be that argon is *not* enhanced at preferentially higher altitudes in the MSE frame (meaning the argon at high altitude in the -E hemisphere is random). We calculated the chi-square value for the -E/+E argon density ratio at 250-300 km and 250-400 km to be 26.94 with 12 degrees of freedom (Df). The corresponding p-value, the probability of exceeding the critical value, is 0.0079. P-values are the probability of a larger value than the χ^2 value; p-values of *less* than 0.05 are considered statistically significant so in this case, we calculate that there is a ~0.79% chance that the null hypothesis (argon density is coincidentally higher in the -E hemisphere) is incorrect. In other words, the chi-square test indicates that the hypothesis, that argon is enhanced at higher altitudes in the MSE frame due to sputtering, is statistically likely.
2. **Spearman's rank-order correlation test**: a non-parametric test that measures the monotonic strength and direction of association between two ranked variables or groups with a correlation coefficient between -1 to 1. Where +/-1.0 is a strong positive and negative correlation, respectively and 0 is no correlation. We calculated the Spearman correlation coefficient for the -E/+E argon density ratio for 250-300km and 350-400km to be -0.044. This indicates that in the MSE frame, the argon densities at 250-300km are not correlated with the argon densities at 350-400km. Put physically, this means that the densities observed at higher altitudes in the MSE frame are likely not to be caused by the same thing as those at 250-300km, which is consistent with sputtering.
3. **Mann-Whitney U test**: a non-parametric test to assess whether two sampled variables or groups are likely to derive from the same population; specifically, it determines whether two populations can be described by the same shape or distribution. We calculated a U value for the -E/+E argon density ratio to be 47. For a confidence level of 0.05, if the U value is less than the critical value, which for this dataset is 59, then we can reject we can reject the null hypothesis that the two groups are from the same population. Therefore we can accept the hypothesis that the argon abundance at the low and high altitudes in MSE coordinates is from a different physical population.

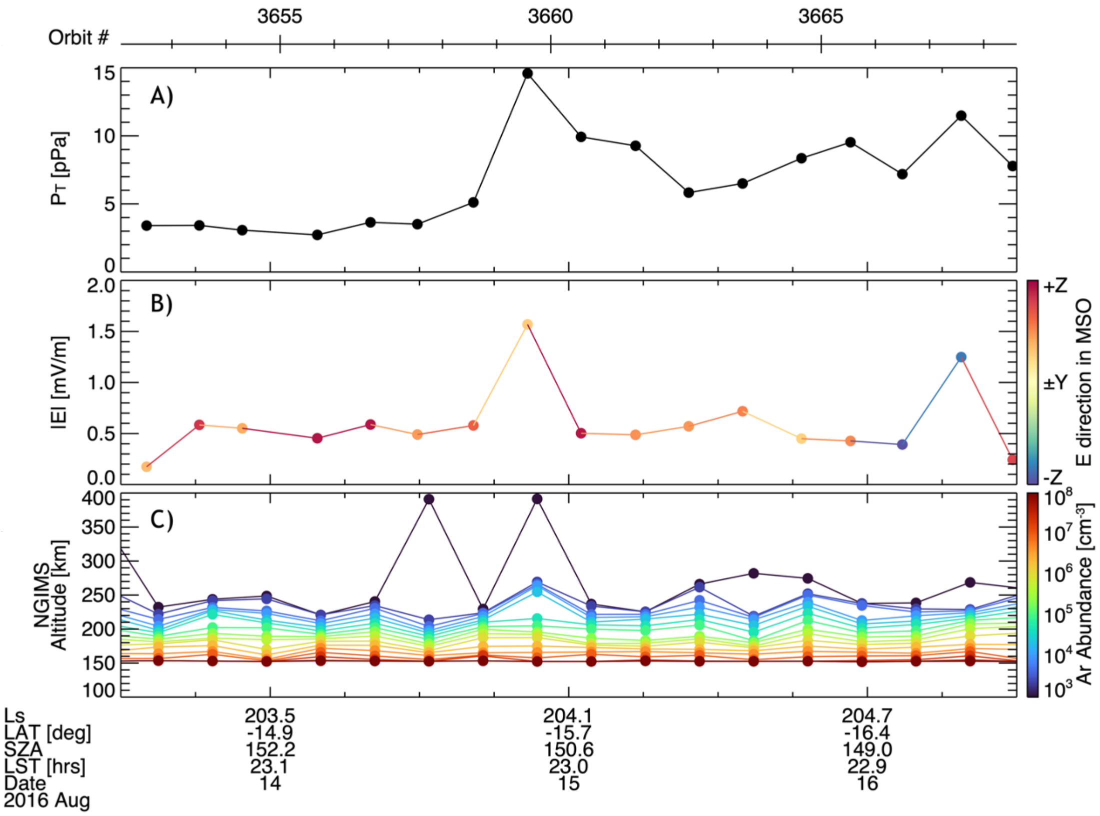

Fig. S1. A case study of atmospheric sputtering occurring during an ICME. (a) Upstream solar wind total perpendicular pressure [pPa], (b) electric field magnitude [mV/m] color coded by vector direction, and (c) the ^{40}Ar abundance altitude profile [cc]. The horizontal bar on the top illustrates the orbit number and dashed line marks the arrival of the ICME shock.

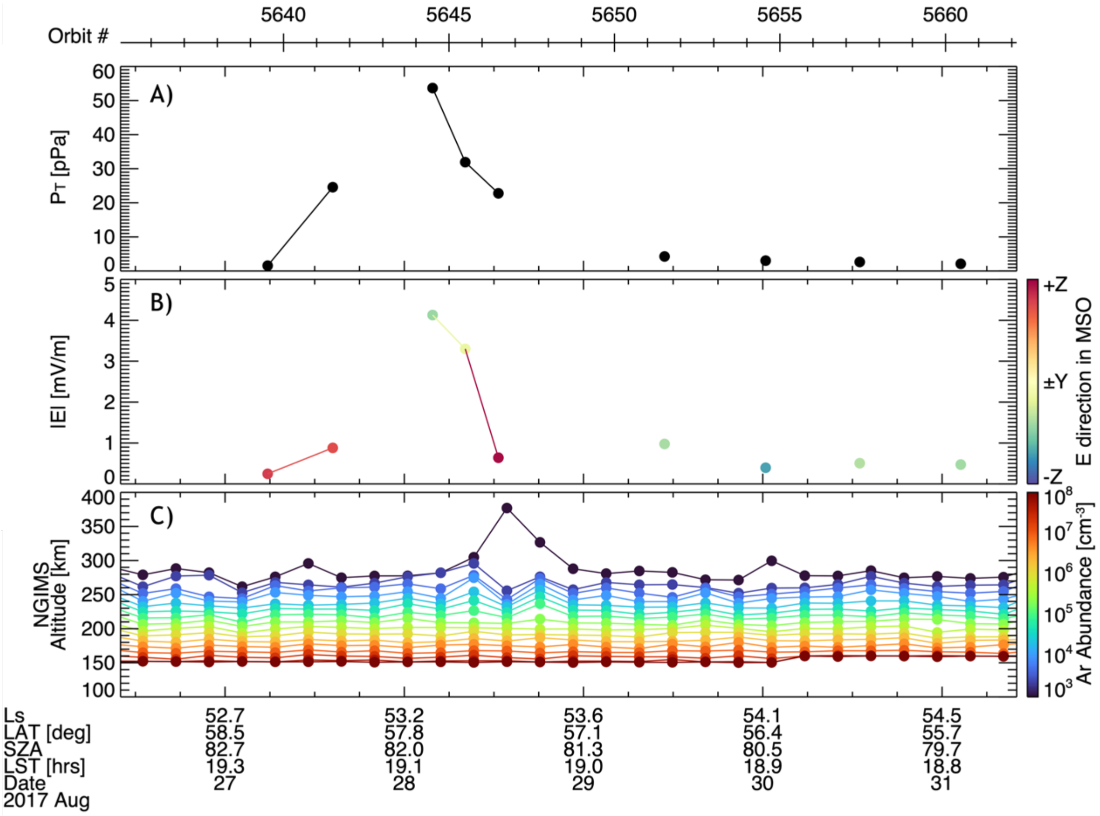

Fig. S2. A case study of atmospheric sputtering occurring during an ICME. (a) Upstream solar wind total perpendicular pressure [pPa], (b) electric field magnitude [mV/m] color coded by vector direction, and (c) the ^{40}Ar abundance altitude profile [cc]. The horizontal bar on the top illustrates the orbit number and dashed line marks the arrival of the ICME shock.
